# Cord Blood Platelet Rich Plasma Derivatives for Clinical Applications in Non-transfusion Medicine

**DOI:** 10.3389/fimmu.2020.00942

**Published:** 2020-05-27

**Authors:** Dinara Samarkanova, Steven Cox, Diana Hernandez, Luciano Rodriguez, Ricardo P. Casaroli-Marano, Alejandro Madrigal, Sergio Querol

**Affiliations:** ^1^Banc de Sang i Teixits, Barcelona, Spain; ^2^Transfusional Medicine Research Group, Vall d'Hebron Research Institute (VHIR), Barcelona, Spain; ^3^Department of Medicine, Universidad Autónoma de Barcelona (UAB), Barcelona, Spain; ^4^Anthony Nolan Research Institute, Royal Free Hospital, London, United Kingdom; ^5^UCL Cancer Institute, Royal Free Hospital, London, United Kingdom; ^6^Department of Surgery, School of Medicine & Hospital Clinic de Barcelona, University of Barcelona, Barcelona, Spain

**Keywords:** cord blood, platelets rich plasma, plasma, platelets, ocular surface disease, skin ulcer, immunomodulation

## Abstract

Cord blood platelet rich plasma (CB-PRP) derivatives have been investigated as potential therapeutic agents for the treatment of diverse conditions including ocular surface disease and skin ulcers. We have developed processes for the formulation of several CB-PRP preparations, which have different composition and attributes. Here we describe the molecular characteristics of these preparations and we make recommendations as to their most appropriate clinical application based on functional and immunomodulatory profiles. We show that incubation of adult peripheral blood mononuclear cells (PBMCs) with all three preparations dramatically reduced the production of INFγ and the expression of NKG2D and CD107a in NK, NKT, and T cells thus diminishing their activation, we propose that the likely mechanism is the high levels of soluble NKG2D ligands present in plasma. Of the three preparations we investigated, CB platelet lysate (PL) and platelet releaseate (PR) have higher concentrations of trophic and pro-angiogenic factors, CB platelet poor plasma (PPP) has the lowest concentration of all analytes measured. Based on these finding we propose that CB-PR is the most suitable raw material for skin wound patches, while CB-PL and PPP can be used to prepare eye drops for severe ocular surface pathologies and inflammatory conditions such as corneal ulcers or severe dry eye disease, respectively.

## Introduction

Human platelet rich plasma (PRP) has been recognized as a rich source of trophic factors (1, 2), which aid healing in several indications including some ocular surface disorders (3, 4), such as severe dry eye disease and corneal ulcers, skin wounds (5) and orthopedic applications. Initially, autologous PRP was used to treat corneal injuries (6) but more recently allogeneic preparations have been favored (7–9). PRP can be obtained readily from both adult peripheral and cord blood (CB). Comparisons of PRP obtained from these two sources suggest that they differ both in the type and in the amount of factors they contain (10). These and other studies argue that CB-PRP has therapeutic advantages over adult PRP as the latter contains more pro-inflammatory molecules which can aggravate certain conditions, while CB-PRP contains higher levels of anti-inflammatory molecules [reviewed in (11)].

CB-PRP contains soluble molecules of NK Group 2, member D (NKG2D) ligands (sNKG2DL), which are in part responsible for NK, NKT, and T cells suppression and may represent a mechanism of fetomaternal tolerance (12). Further work elucidated the differential roles of the various types of sNKG2DLs and revealed soluble unique long 16 binding proteins (sULBP1) as the most abundant soluble ligand in CB-PRP that directly downmodulated NK cell cytotoxicity in a dose-dependent manner (13). Additionally, we showed that polymorphisms in Major Histocompatibility Complex Class I-related Chain A (MICA) which encode either valine (Val) or methionine (Met) at residue 129 affected the ability of soluble MICA (sMICA) to suppress NK cell function. Met is known to bind with strong affinity to NKG2D whereas Val has weaker binding (14).

Depending on the processing protocols used to separate the PRP from whole blood, a variety of preparations can be made which have different properties. An initial low speed centrifugation of whole CB units yields CB-PRP which can be further processed. CB-PRP contains a variety of growth factors (GF), cytokines and other immunomodulatory factors that can act on the proliferation, differentiation and function of both immune and other cells such as fibroblasts. CB-PRP also contains albumin, transferrin, fibronectin, fibrinogen, minerals, adiponectin, vitamin A and E, several fatty acids and platelets (15), which contribute as trophic factors in wound healing mechanisms of epithelial cells of the cornea and its stromal tissue (16). These elements have also been implicated in cutaneous scarring formation (17). Thus, the availability through CB banking and the therapeutic benefits of the factors contained in CB-PRP derived products make CB an ideal product source for the treatment of a variety of diseases and inflammatory conditions. These benefits can be enhanced by processing the PRP both by concentration of the platelets and their lysis, which releases platelet-derived growth factor (PDGF), basic fibroblast growth factor (bFGF), epithelial growth factor (EGF), vascular endothelial growth factor (VEGF), and transforming growth factor beta (TGFβ) among others, all of which have been shown to enhance tissue regeneration (18).

The purpose of this study was to compare the profile of GFs, cytokine and immune elements of CB-PRP derived preparations to determine which ones would be best suited for clinical applications. We formulated, from CB-PRP, three different preparations: (a) platelet poor plasma (CB-PPP), (b) platelet concentrate lysate (CB-PL), and (c) platelet concentrate releaseate (CB-PR).

The concentrations of various GFs, cytokines and soluble NKG2D ligands, together with their immunomodulatory effects on Natural Killer (NK) cells and CD8^+^ T cells were determined in the different CB-PRP preparations. Taken together, the results can help us determine the optimal CB-PRP derived preparations for different clinical applications for ocular surface diseases and skin wound healing.

## Materials and Methods

### Ethical Considerations

PRP derived samples from individual CB units were obtained from BioBank (BST). The informed consent for the use of samples for research was obtained in accordance with the requirements of the Declaration of Helsinki and local laws at the time of collection. This study was approved by the BST local Research Ethics Committees (HCB/2017/0785).

### CB Unit Collection

CB units were collected in authorized maternity hospitals. Before delivery, mothers signed an informed consent for donation that allows the use of these samples for research and validation purposes. Qualified health care professionals collected CB units while placenta was still *in utero* using validated procedures (19). The collection bag contains 25 mL of citrate phosphate dextrose (CPD) as anticoagulant.

### CB Unit Verification

Full serological and microbiological analysis of the CB units was carried out to exclude infection. Tests were performed for the presence of Hepatitis A, B, and C viruses, human immunodeficiency virus, *Treponema Pallidum* (Syphilis), *Trypanosoma cruzi* (Chagas disease), and cytomegalovirus as well as aerobic and anaerobic bacterial and fungi (BacTAlert, Biomerieux, INC. Durham). Quarantine was applied as a minimum for 2 weeks. Only units, which were non-reactive, were further processed.

### Preparation of CB-PRP Derivatives

**Figure 1:**
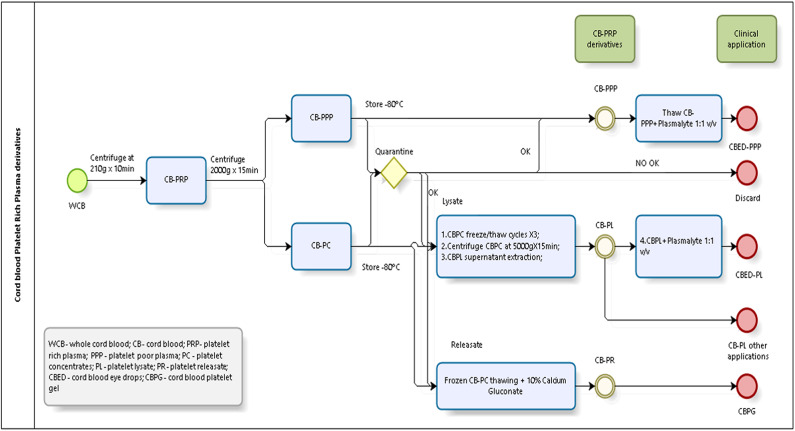
CB-PRP derivatives processing scheme.

Briefly, a total of 20 whole CB (WCB) units were used for the experiments. Units were treated individually and from each unit we derived all 3 preparations described. Each unit was centrifuged at low speed (210 g) for 10 min, the supernatant collected constitutes the PRP. The PRP was then centrifuged at high speed (2,000 g) for 15 min to obtain two fractions, a PPP and a platelet pellet, which was resuspended in PPP to obtain a platelet concentrate (CB-PC) in range of 800–1,200 × 10^9^ platelets/L in 10 (±5) mL and then stored at −80°C. CB-PC fraction was frozen for quarantine. Only microbiology and serology results negative samples of CBPC were used for preparation of platelet lysate (CB-PL) and platelet releaseate (CB-PR). Figure 1 shows a schematic of the processing of CB to obtain the different preparations tested.

#### CB-PPP Preparation

The remaining PPP was frozen at −80°C for future analysis; this product is CB-PPP.

#### CB-PL Preparation

CB-PC samples underwent 3 freeze (~-80°C)/thaw (~37°C) cycles (20) to lyse platelets and release GFs followed by a centrifugation step at 5,000 g for 15 min. The collected GF rich supernatant free of intact platelets was subsequently stored at −80°C for future use. This CB-PL, diluted with Plasmalyte (Baxter, USA) (1:1, v:v) is used to prepare CB eye drop (CBED), which was used in clinical trial [ClinicalTrails.gov ID NCT03084861]. For this study was used only CB-PL, without dilution as the active ingredient.

#### CB-PR Preparation

CB-PC underwent one freeze/thaw cycle (which in our experience is insufficient to cause major platelet lysis and allows the storage of this fraction ready for calcium gluconate activation), the resulting fraction termed platelet releasate was treated with 10% calcium gluconate (Braun®, Terrassa, Spain) (1:10, v:v) and incubated for 1 h at room temperature. This jellification step forms the basis for the clinical skin patch, which has been used for clinical trial (ClinicalTrails.gov ID NCT02389010). For the purpose of the analyte measurement in this study, the releasate was also treated with 10% calcium gluconate (as above) but in the presence of heparin at 0.61 IU/mL to prevent clotting and thus the jellification of the plasma and trapping GFs, which would have confounded the measurements of factors in this preparation. After a centrifugation step (5,000 g for 15 min) the supernatant free of intact platelets (checked by automatic counter-Beckman-Coulter) was used for analyte measurements or stored at −80°C until needed.

### CB-PRP Derived Products Cytokines and GF Quantification

CB-PRP preparations of CB-PPP, CB-PL, and CB-PR from single units were analyzed using magnetic beads of Luminex kits (R&D Systems, Abingdon, UK) according to manufacturer's recommendations, to determine concentration of following analytes: (plate 1) PDGF AB/BB, EGF, bFGF, VEGF, IL1/6/10, TNFα; (plate 2) TGFβ1; (plate 3) HGF; (plate 4) MMP2/9; (plate 5)TIMP1-4.

### PBMC Functional Analysis

Peripheral blood mononuclear cells (PBMCs) were isolated from whole blood (WB) from consenting, healthy adult male or female volunteer donors by density-gradient centrifugation using Lympholyte®-H solution (Cedarlane, ON, Canada). PBMCs were then used for co-culture with the various CB-PRP derived preparations for functional analysis. For assessment of cellular function of CD3- CD56bright/dim NK cells, CD3+ CD56+ NKT cell and CD3+ CD56– T cells, healthy adult donor PBMCs were plated in RPMI (Lonza, Slough, UK) containing 10% heat-inactivated fetal calf serum (FCS) supplemented with 1% penicillin and streptomycin (complete media) and containing human IL-2. Test cultures using CB-PRP preparations: CB-PPP, CB-PL or CB-PR were diluted with an equal volume of complete media containing 200 IU IL-2 per well (200 μl volume) in 96-well U-bottom plates. Results were normalized against PBMCs cultured with complete media containing 200 IU IL-2 per well and expressed as percentage of maximum expression (for measurement of NKG2D, CD107a or IFN-γ expression). All cultures were incubated at 37°C with 5% CO_2_ for 48 h before Phorbol 12-myristate 13-acetate (PMA) and ionomycin stimulation. Duplicate cultures were carried out for each experiment without PMA and ionomycin stimulation to assess levels of NKG2D and baseline CD107a expression. NKG2D expression on the relevant cells was determined using the unstimulated cultures and CD107a background levels in unstimulated cultures were subtracted from the values obtained for equivalent stimulated cells. Three independent experiments were carried out using four different, healthy PBMC donors, each tested with CB-PPP, CB-PL, CB-PR, or media only and data points represent donor means for each replicate test sample.

### Flow Cytometry

Briefly, cells were labeled with fluorochrome-conjugated antibodies in Phosphate-buffered saline (PBS) with Bovine Serum Albumin (BSA) (0.5%) for 10 min at 4°C. Antibodies (BD Biosciences, Oxford, UK) were as follows: CD3 (SK7), CD56 (B159), CD107a (HA4A3), NKG2D (BAT221, Miltenyi Biotec, Bisley, UK). Viability was assessed using Annexin V and 7-AAD. For quantitation of CD107a, cells were re-suspended in complete media containing 100 ng/ml PMA, 1 μg ionomycin and 0.1% 2-mercaptoethanol (stimulated) or complete media with 0.1% 2-mercaptoethanol (non-stimulated) for 2 h at 37°C. Fluorescence minus one (FMO) controls (where samples are stained sequentially with all antibodies except one) were used to set gates. Acquisition was performed using a Fortessa II flow cytometer (BD Biosciences, Oxford, UK) and analysis was carried out using FlowJo version 10.5.0 (Tree Star Inc., OR, USA). The gating strategy used for analysis of lymphocyte subtypes was performed as described in our previously study (12) and is shown in Supplementary Figure S1.

### Soluble NKG2DL and IFN-γ Detection and Quantification Assays

Soluble MICA/B (DY1300/DY1599) and ULBP1 (DY1380) were quantified in CB-PRP derivatives directly and IFN-γ (DY285) was measured in PBMC stimulated supernatants using Duoset ELISA kits (R&D Systems, Abingdon, UK), according to manufacturer's instructions.

### MICA-129 (Valine or Methionine) SNP Genotyping

Genomic DNA for MICA genotyping was obtained from WCB using QIAamp® DNA blood minikit (Qiagen, GmbH, Hilden, Germany). The SNP rs1051792 (G/A) causing substitution of *Val* (G) for *Met* (A) at position 129 of the MICA gene was genotyped using a TaqMan® assay (Applied Biosystems, Foster City, CA, USA). PCR amplification conditions were as recommended by the manufacturer and reactions contained the forward primer 5′-GCTCTTCCTCTCCCAAAACCT-3′ and reverse primer 5′-CGTTCATGGCCAAGGTCTGA-3′ and the two allele-specific dye labeled probes FAM-5′-AATGGACAGTGCCCC-3′ and VIC-5′-AATGGACAATGCCCC-3′. Results were obtained and interpreted using the CF96 real-time PCR system (Biorad, Hercules, CA, USA). Confirmatory typing was performed using DNA extracted from WCB and polymerase chain reaction (PCR) amplification and sequencing of exon 3 of the MICA gene. Results were used to determine the presence of “A,” “G” or both encoding *Met* or *Val* at residue 129 (ATG or GTG, respectively). Exons 2–6 of the MICA gene were amplified as previously described (21) using a forward primer located in intron 1 (5′- CACCTGTGATTTCCTCTTCCCCAGAGC-3′) and reverse primer in the 3′ untranslated region (5′-CTAACAATTTGCAGCMTCCAACAAC-3′). Cycle sequencing was performed using standard protocols and exon 3 forward sequencing primer (5′- CCCTGGGCTGAGTTCCTC-3′) and reverse sequencing primer (5′- ATAGCACAGGGAGGGTTT-3′).

### Statistical Analysis

Results are shown as mean with standard error of the mean (SEM) and were evaluated using Graphpad Prism 8 (Graphpad Software, CA, USA. Datasets were analyzed using both paired and unpaired non-parametric statistical analysis as the sample size is too small to assume normal distributions. As we had 3 categories of sample, we used Friedmans's test for the paired comparisons and a Kruskal-Wallis for the unpaired analysis. Significance levels are indicated as *p*< *0.05* (^*^), *p*< *0.01* (^**^), *p*< *0.001* (^***^), and *p* < 0.0001 (^****^), unless the exact *p*-value is given. For comparisons between genotypes only we used Mann-Whitney.

## Results

### Comparison of Cytokine and GF Concentration Among the Different Preparations of CB-PRP Derivatives

The prepared CB-PC units contained 1,004 ± 76 × 10^9^ platelets (PLT)/L (*n* = 10) and were within the standard range of 800–1,200 × 10^9^ PLT/L, which was used to prepare CB-PL and CB-PR.

**Figure 2:**
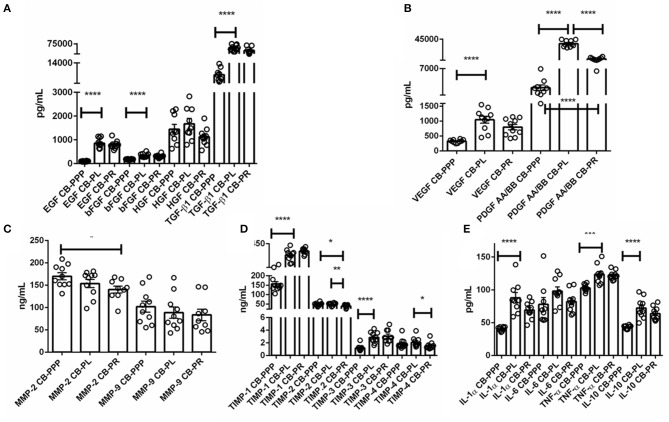
**(A)** Trophic and wound healing factors in CB-PRP preparations in pg/mL, growth factors analyzed were EGF, bFGF, HGF, and TGF-β1. **(B–D)** Concentration of the angiogeneic group of factors in CB-PRP derived preparations. Results show concentration in pg/mL and ng/mL of each factor. The analytes measured were **(B)** VEGF and PDGF in pg/mL; **(C)** MMP-2 and MMP-9 in ng/mL; **(D)** TIMP1-4 in ng/mL. **(E)** Concentration of inflammatory and anti-inflammatory cytokine group of analytes in CB-PRP preparations. Data points represent concentration mean of IL-1α, IL-6, and TNF-α pro-inflammatory and IL-10 anti-inflammatory cytokines. CB-PRP preparations investigated were, cord blood platelet poor plasma (CB-PPP; *n* = 10), cord blood platelet lysate (CB-PL; *n* = 10) or cord blood platelet releasate (CB-PR; *n* = 10). Each sample was measured in duplicate in two separate experiments. Data points represent concentration mean. Statistical analysis was performed using non-parametric one-way ANOVA (Kruskal-Wallis test with Dunn's *post-hoc* test for unpaired samples and Friedman's for paired samples). *p < 0.05, **p < 0.01, ***p < 0.001, ****p < 0.0001.

The levels of trophic and wound healing factors (group I), pro-angiogenic molecules (group II), pro-inflammatory (group III.A) and anti-inflammatory (group III.B) GFs and cytokines for all three preparations were analyzed and compared, the results are shown in Table 1, Figure 2, we have included statistical analysis of results both for paired and unpaired samples on the table, but the graph shows only significance for the paired samples. For comparative purposes the levels in tears of some of these factors are as follows: EGF (200–3,000 pg/mL), TGFβ (2–10 ng/mL), HGF (200–500 pg/mL), VEGF (2–19 pg/mL), PDGF (90–1,700 pg/mL) (3). In CB-PL preparations the concentration of most factors tested was higher than that observed in CB-PPP, however this did not reach significance in HGF, MMP-2, TIMP-1, 2, and 4 or IL6 and was lower for MMP-9. There were no statistically significant differences in concentration in any of the analytes examined between CB-PR and CB-PL, except for HGF, MMP-2, and TIMP-2 and 4, which were higher in CB-PL (Table 1, Figure 2).

**Table 1:** Comparative composition of CB-PRP derivatives.

			**Paired**			**Unpaired**		
**Analyte**	**Sample**	**Concentration mean ± SEM**	***p*-Value CB-PPP vs. CB-PL**	***p*-Value CB-PPP vs. CB-PR**	***p*-Value CB-PR vs. CB-PL**	***p*-Value CB-PPP vs. CB-PL**	***p*-Value CB-PPP vs. CB-PR**	***p*-Value CB-PR vs. CB-PL**
**Group I (trophic and wound healing factors)**								
		**pg/mL**						
EGF	CB-PPP	93.5 ± 5	0.0004	0.0110	>0.9999	0.0002	0.008	>0.9999
	CB-PL	858 ± 66						
	CB-PR	800.6 ± 54						
bFGF	CB-PPP	179.5 ± 5	0.0004	0.0110	0.9999	0.0001	0.0011	>0.9999
	CB-PL	348 ± 26						
	CB-PR	310 ± 21						
HGF	CB-PPP	1446 ± 204	0.0553	0.29669	0.0002	>0.9999	0.6702	0.1399
	CB-PL	1676 ± 227						
	CB-PR	1127 ± 116						
TGF-β1	CB-PPP	8767 ± 929	0.0002	0.1365	0.1365	0.0003	0.0126	0.8665
	CB-PL	58646 ± 3553						
	CB-PR	49461 ± 3949						
**Group II (pro-angiogenic factors)**								
		**pg/mL**						
VEGF	CB-PPP	329 ± 17	<0.0001	0.0760	0.0760	<0.0001	0.0038	0.7279
	CB-PL	1046 ± 367						
	CB-PR	798 ± 90						
PDGF AB/BB	CB-PPP	3388 ± 535.5	<0.0001	0.1017	0.1017	<0.0001	0.0569	0.0447
	CB-PL	37267 ± 1817						
	CB-PR	10276 ± 752						
		**ng/mL**						
MMP-2	CB-PPP	170 ± 8	>0.9999	0.0012	0.0140	>0.9999	0.0905	0.1607
	CB-PL	153 ± 9						
	CB-PR	140 ± 7.7						
MMP-9	CB-PPP	101 ± 12	0.0286	0.0005	0.7158	>0.9999	0.8959	>0.9999
	CB-PL	88 ± 12						
	CB-PR	83.4 ± 13						
TIMP-1	CB-PPP	157 ± 12	0.0733	0.0005	0.4008	0.0100	0.0003	>0.9999
	CB-PL	332 ± 18						
	CB-PR	372 ± 11						
TIMP-2	CB-PPP	48 ± 2.6	0.2969	0.0553	0.0002	0.8156	0.0720	0.0024
	CB-PL	48.7 ± 2						
	CB-PR	38.8 ± 2.5						
TIMP-3	CB-PPP	1.1 ± 0.01	0.0029	0.0065	>0.9999	0.0019	0.0033	>0.9999
	CB-PL	2.8 ± 0.2						
	CB-PR	3.0 ± 0.3						
TIMP-4	CB-PPP	1.8 ± 0.2	0.2969	0.0553	0.0002	0.8156	0.7047	0.0667
	CB-PL	2.0 ± 0.2						
	CB-PR	1.5 ± 0.2						
**Group III.A (pro-inflammatory GFs and cytokines)**								
		**pg/mL**						
IL-6	CB-PPP	77 ± 10	0.0140	>0.9999	0.1017	0.0778	>0.9999	0.4621
	CB-PL	98 ± 6						
	CB-PR	82 ± 5						
IL-1α	CB-PPP	40.5 ± 1.0	<0.0001	0.1017	0.1017	<0.0001	0.0081	0.6702
	CB-PL	88 ± 8						
	CB-PR	69 ± 4						
TNF-α	CB-PPP	103 ± 1.6	0.0024	0.0024	>0.9999	0.0008	0.0009	>0.9999
	CB-PL	123.6 ± 4						
	CB-PR	122 ± 2						
**Group III.B (anti-inflammatory cytokines)**								
		**pg/mL**						
IL-10	CB-PPP	43.5 ± 0.8	0.0004	0.0110	>0.9999	<0.0001	0.0020	>0.9999
	CB-PL	72 ± 8						
	CB-PR	63.8 ± 3						

*Non-parametric comparison test. n = 10/product type*.

As expected, for processed CB-PPP samples, that had 8 ± 3 × 10^9^ PLT/L in 24 ± 4 mL (*n* = 10), all measured analytes had lower concentrations, with the exception for HGF, TIMP-2 and 4 which were no different and MMP-2 and 9, which were higher in CB-PPP than in CB-PR (Table 1). Pro-angiogenic factors showed low concentrations, except for MMP-9. Both pro and anti-inflammatory cytokines have significantly higher concentration in CB-PL than CB-PPP, but are similar between CB-PL and CB-PR (Table 1, Figure 2). In adult serum the concentrations of these factors are as follows: EGF (500–1,000 pg/mL), TGFβ (6–50 ng/mL), HGF (100–1,000 pg/mL), VEGF (1,000–5,000 pg/mL), PDGF (30–100 ng/mL) (3). While levels of EGF, VEGF, HGF are comparable between CB-PL, CB-PR, and adult serum, the concentration of PDGF is lower in CB-PR. TGFβ is slightly higher in CB-PC derived products and comparable to adult serum levels in CB-PPP samples.

### Effect of CB-PRP Preparations on Donor Cell Viability

After measuring the levels of GFs and other factors, we investigated the effect of the PRP derivatives preparations on immune cells, specifically on NK cells. We and others have established that the immunomodulatory effects of plasma leading to suppression of NK cells are exerted through both the high concentration of TGFβ and NKG2D engagement with sNKG2DLs. Therefore, we examined the effects of the different preparations on the phenotype and activity of NK, NKT, and T cells.

**Figure 3:**
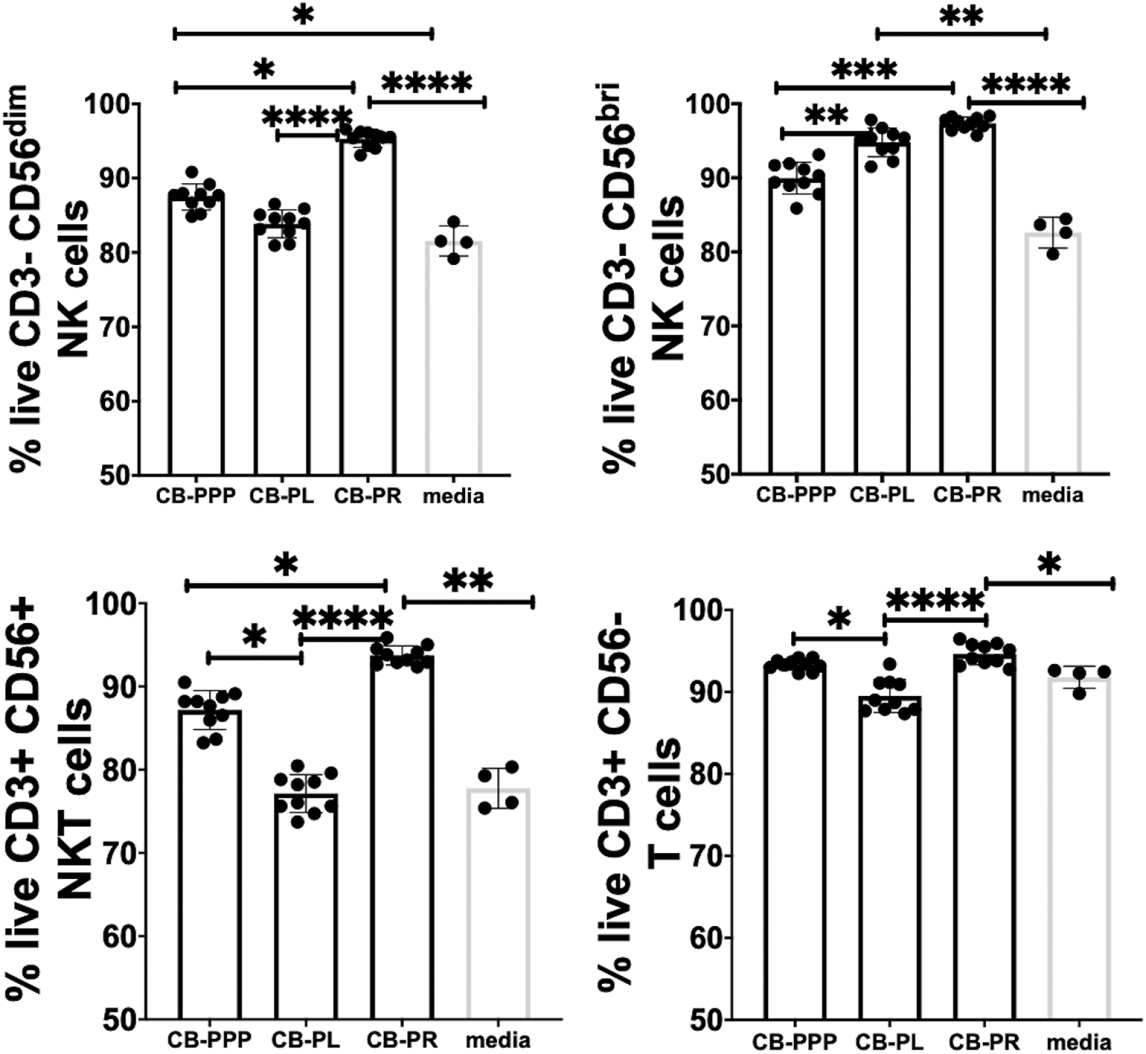
Viability of CD3- CD56 dim, CD3- CD56 bright NK cells, CD3+ CD56+ NKT cells, and CD3+ CD56- T cells from adult donor PBMCs (*n* = 3) after incubation with cord blood plasma preparations or complete media only. Results show percentage of live cells (Annexin V negative, 7-AAD negative) for each cell type. Cord blood plasma preparations investigated were cord blood platelet poor plasma (CB-PPP; *n* = 10), cord blood platelet lysate (CB-PL; *n* = 10), or cord blood platelet releasate (CB-PR; *n* = 10). PBMCs were incubated with each preparation diluted 50:50 with media and supplemented with IL-2, or with complete media and IL-2 only for 48 h prior to antibody staining and flow cytometry analysis. Each experiment was repeated with four different PBMC donors and data points represent donor means. Statistical analysis was performed using non-parametric one-way ANOVA (Kruskal-Wallis test with Dunn's *post-hoc* test for unpaired samples and Friedman's for paired samples). *p < 0.05, **p < 0.01, ***p < 0.001, ****p < 0.0001.

Initially we examined the effect of incubation of PBMCs in the presence of the different preparations to ensure they had no toxic effects and viability was comparable. We incubated the cells in standard cell culture conditions, with 50% solution of each PRP preparation, diluted with complete media containing IL-2. We then determined the percentage of live lymphocytes by gating CD3-CD56 bright/dim NK cells, CD3+ CD56+ NKT cells and CD3+ CD56– T cells cultured with each of the CB-PRP preparations. The results, shown in Figure 3. show a general improvement in viability when cells were cultured with CB-PRP-supplemented media compared to media only. For all cell types investigated, viability was significantly improved in the presence of CB-PR compared to media only. While the presence of CB-PL was only significantly better compared to media alone in CD3–CD56 bright NK cells, CB-PPP was significantly better only in CD3-DC56 dim NK cells.

The viability of CD3– CD56 bright NK cells was not significantly different between cells grown in CB-PR or CB-PL when samples were compared either paired or unpaired. Growing them in the presence of CB-PL or CB-PR increased the viability significantly compared to CB-PPP, again regardless of samples being paired or unpaired. For CD3– CD56 dim NK cells the presence of CB-PR improved viability significantly compared to CB-PPP and CB-PL again regardless of sample pairing, and there was no significant difference between CB-PPP and CB-PL in these cells. The viability of CD3+ CD56+ NKT was highest in cells grown in CB-PR but the difference was only slightly significant between CB-PR and CB-PL when samples were analyzed paired. For CD3+CD56– T cells, again CB-PR is significantly better than CB-PL, but no different than CB-PPP whether samples are analyzed paired or unpaired.

Overall, incubation of PBMCs in the presence of all three CB-PRP preparations had a beneficial effect on viability and no detrimental or toxic effect.

### Incubation With CB-PPP, CB-PL, and CB-PR Shows Differential Reduction in NKG2D Expression

**Figure 4:**
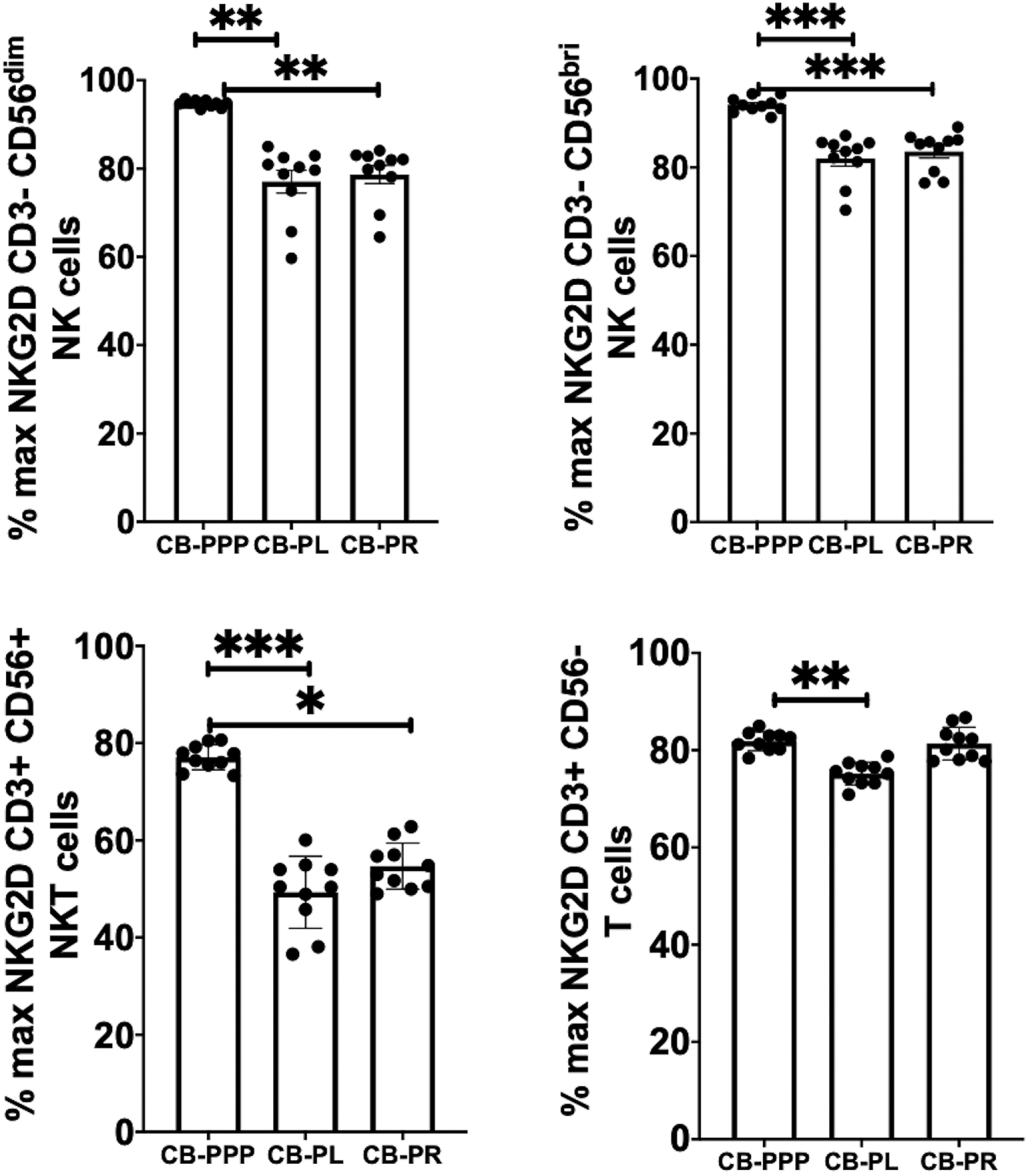
Expression of NKG2D on NK, NKT and T cells from healthy donors is reduced by incubation with, CB-PPP, CB-PL, and CB-PR. Data represents analysis of CD3– CD56 dim, CD3– CD56 bright NK cells, CD3+ CD56+ NKT cells and CD3+ CD56– T cells in adult donor PBMCs (*n* = 4) after incubation with CB-PRP preparations or complete media only. Results show percentage of maximum expression relative to complete media for each cell type from unstimulated cultures. CB-PRP preparations investigated were cord blood platelet poor plasma (CB-PPP; *n* = 10), cord blood platelet lysate (CB-PL; *n* = 10), or cord blood platelet releasate (CB-PR; *n* = 10). PBMCs were incubated with each preparation diluted 50:50 with media and supplemented with IL-2, or with complete media and IL-2 only for 48 h prior to antibody staining and flow cytometry analysis. Each experiment was repeated with four different PBMC donors and data points represent donor means. Statistical analysis was performed using non-parametric one-way ANOVA (Kruskal-Wallis test with Dunn's *post-hoc* test for unpaired samples and Friedman's for paired samples). *p < 0.05, **p < 0.01, ***p < 0.001.

Generally, incubation of peripheral blood CD3– CD56 dim and bright NK cells, CD3+ CD56+ NKT cells and CD3+ CD56– T cells with CB-PRP derivatives resulted in reduced expression of NKG2D relative to media only (Figure 4). Significant differences were observed between the different preparations of CB-PRP with all cell types, with CB-PL reducing NKG2D expression the most. While incubation with CB-PPP only reduced NKG2D expression to 94.7% ± 0.25 of maximal expression in CD3– CD56 dim NK cells, CB-PR reduced it to 78.6% ± 2.05 (*p* = *0.0052*) and CB-PL to 77.04% ± 2.57 (*p* = *0.0010*). Similar NKG2D expression patterns were seen in CD3– CD56 bright NK cells after incubation with the 3 preparations. Analysis of CD3+ CD56+ NKT cells showed a similar reduction in NKG2D expression after treatment with both CB-PL and CB-PR (49.3% ± 2.3% and 54.7% ± 1.5%, respectively) which was significantly more pronounced than culture with CB-PPP (*p* = *0.0004* and *p* = *0.0110*, respectively in paired analysis). A similar profile to CD3+ CD56+ NKT cells was observed for CD3+ CD56– T cells, though the overall reduction in expression was less dramatic.

### Incubation With CB-PL, CB-PPP, and CB-PR Reduces Expression of CD107a and IFN-γ

**Figure 5:**
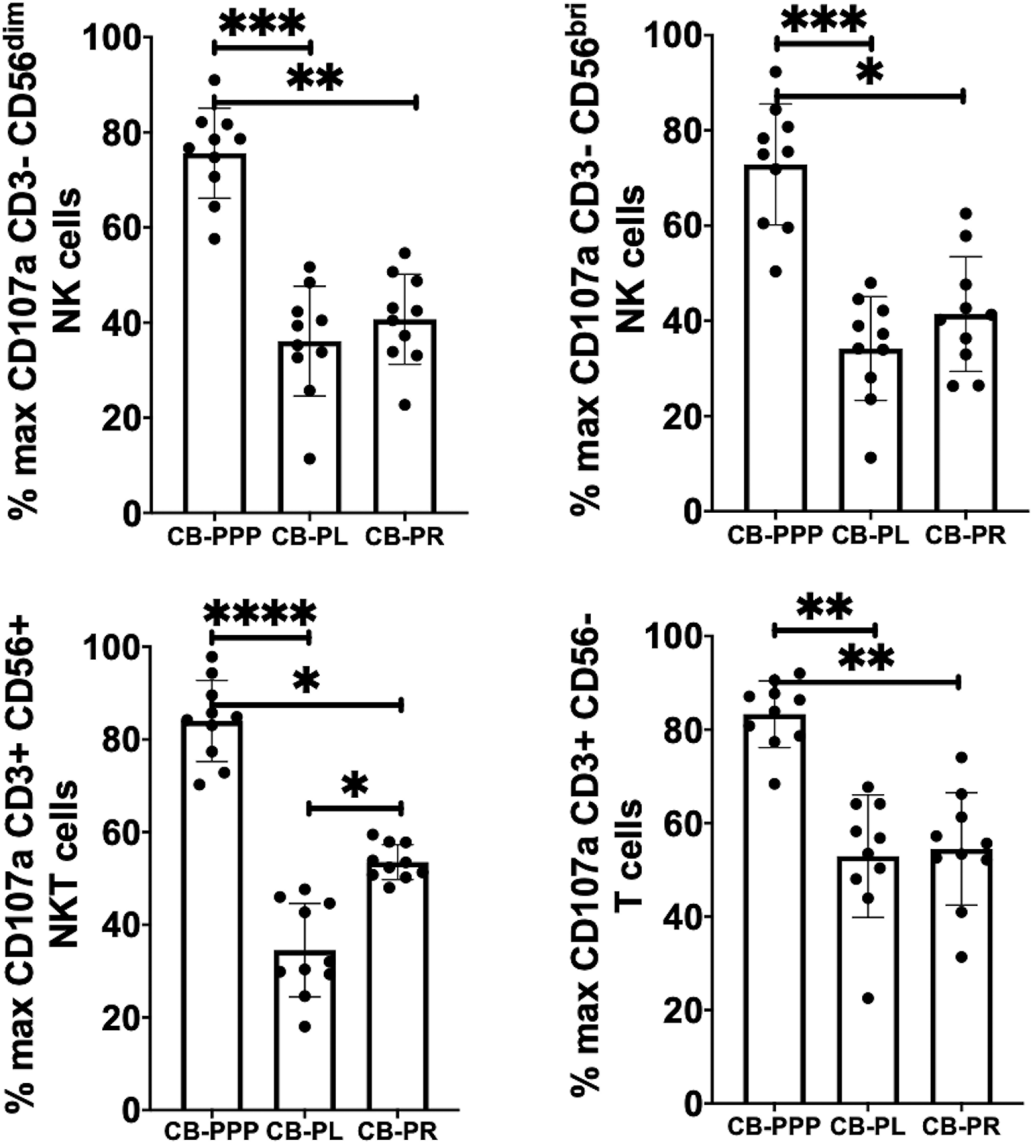
Expression of CD107a after PMA and ionomycin stimulation on NK, NKT, and T cells from healthy donors is reduced by incubation with, CB-PPP, CB-PL, and CB-PR. Data represents analysis of CD3– CD56 dim, CD3– CD56 bright NK cells, CD3+ CD56+ NKT cells and CD3+ CD56– T cells in adult donor PBMCs (*n* = 4) after incubation with CB-PRP derived samples or complete media only. Results show percentage of maximum expression relative to complete media for each cell type following 2 h stimulation with PMA and ionomycin. CB-PRP preparations investigated were cord blood platelet poor plasma (CB-PPP; *n* = 10), cord blood platelet lysate (CB-PL; *n* = 10), or cord blood platelet releasate (CB-PR; *n* = 10). PBMCs were incubated with each preparation diluted 50:50 with media and supplemented with IL-2, or with complete media and IL-2 only for 48 h prior to antibody staining and flow cytometry analysis. Each experiment was repeated with four different PBMC donors and data points represent donor means. Statistical analysis was performed using non-parametric one-way ANOVA (Kruskal-Wallis test with Dunn's *post-hoc* test for unpaired samples and Friedman's for paired samples). *p < 0.05, **p < 0.01, ***p < 0.001, ****p < 0.0001.

**Figure 6:**
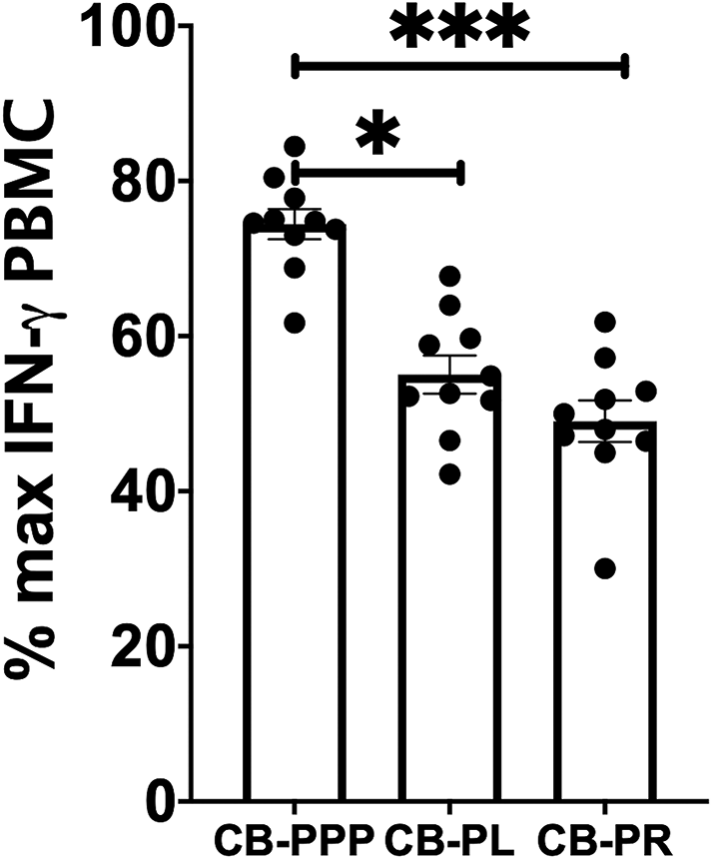
IFN-γ production by PBMCs from healthy donors is reduced by incubation with CB-PPP, CB-PL and CB-PR samples. Results show percentage of maximum IFN-γ expression in culture supernatants relative to complete media following 2 h stimulation with PMA and ionomycin. CB-PRP preparations investigated were cord blood platelet poor plasma (CB-PPP; *n* = 10), cord blood platelet lysate (CB-PL; *n* = 10), or cord blood platelet releasate (CB-PR; *n* = 10). PBMCs were incubated with each preparation diluted 50:50 with media and supplemented with IL-2, or with complete media and IL-2 only for 48 h prior to antibody staining and flow cytometry analysis. Each experiment was repeated with four different PBMC donors and data points represent donor means. Statistical analysis was performed using non-parametric one-way ANOVA (Kruskal-Wallis test with Dunn's *post-hoc* test for unpaired samples and Friedman's for paired samples). No significant differences between the different CB-PRP preparations were found. *p < 0.05, ***p < 0.001.

Incubation of all cell types examined with CB-PL resulted not only in a significant reduction in the expression of NKG2D, but also in a dramatic, and statistically significant, reduction in the expression of CD107a indicating a reduction in activation potential as shown in Figure 5. For example, percentage of maximum expression of CD107a in CD3- CD56bri NK cells incubated with CB-PL was 34.2% ± 3.4 SEM compared to 72.8% ± 4.01 SEM with CB-PPP (*p* = *0.002*) and 41.4% ± 3.81 SEM with CB-PR (*p* = *0.539*). Similar differences were seen in CD3– CD56 dim NK cells and CD3+ CD56+ NKT cells. Compared with NK and NKT cells, a smaller reduction in CD107a expression was seen in CD3+ CD56– T cells and there was no significant difference between CB-PR and CB-PL. However, CD107a expression, in the presence of both CB-PL (52.9% ± 4.14) and CB-PR (54.48 ± 3.8), was significantly reduced compared to incubation with CB-PPP (83.3% ± 2.2) (*p* = *0.0024*). We measured the total IFN-γ produced after stimulation of cultures following the incubation period and results are shown in Figure 6. All CB-PRP preparations resulted in reduced IFN-γ production compared to complete media only, though there were no significant differences between CB-PR and CB-PL, treatment with both these preparations resulted in significantly less IFN-γ production compared to CB-PPP.

### CB-PRP Preparations CB-PL, CB-PPP, and CB-PR Contain sNKG2DLs

**Figure 7:**
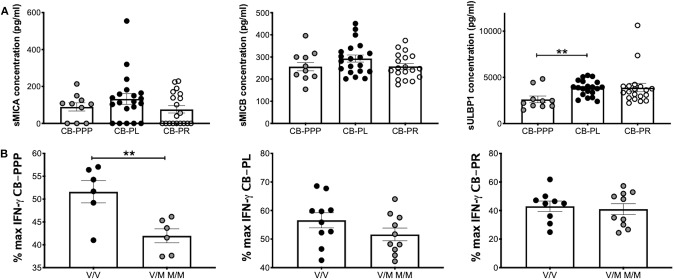
CB-PRP preparations (CB-PPP, CB-PL, and CB-PR) contain immunosuppressive soluble NKG2D ligands. **(A)** ELISA analysis of soluble NKG2D ligand content (sMICA, sMICB, and sULBP1) in CB-PPP (*n* = 10), CB-PL (*n* = 20), and CB-PR (*n* = 19) revealed similar quantities of sMICA and sMICB. Significantly higher concentration of sULBP1 was found in CB-PL compared with CB-PPP. **(B)** MICA genotyping was performed with DNA obtained from whole CB used to prepare CB-PL (*n* = 20), CB-PR (*n* = 19), and CB-PPP (*n* = 12). MICA-129 valine homozygotes (V/V) were compared with heterozygote and homozygote MICA-129 methionine genotypes (V/M or M/M) for each preparation. Statistical analysis was performed using non-parametric one-way ANOVA (Kruskal-Wallis test with Dunn's *post-hoc* test) or Mann Whitney test. **p < 0.01.

We previously reported that CB plasma contains soluble NKG2DLs such a sMICA, sMICB, and sULBP1 (12, 22). Using ELISA, we measured the concentration of these ligands in the different preparations of CB-PRP. For this analysis we used two sets of preparations for CB-PL and CB-PR with a total of 20 and 19 samples, respectively. One set of CB-PPP was too dilute for the limit of detection of the assay, therefore only 10 samples were utilized. We found that sMICA ligands were present in most samples of CB-PPP, CB-PL, and CB-PR and all contained sMICB and sULBP1 (Figure 7A). There was no significant difference in levels of sMICA or sMICB among the different CB-PRP preparations but significantly more sULBP1 could be detected in CB-PL (3.85 ng/ml ± 0.20) compared to CB-PPP (2.62 ng/ml ± 0.35) (*p* = *0.0058*).

### MICA Polymorphisms and Their Effects on IFNγ Production

The MICA polymorphism encoding either valine (Val; V) or methionine (Met; M) at residue 129 of the MICA protein is thought to influence functional differences in NK cell activation (14). We categorized CB-PRP preparations (CB-PL, CB-PPP, and CB-PR) as either Val homozygous (V/V) or homozygous and heterozygous samples containing Met (M/M and V/M) and examined the amount of IFN-γ produced by NK cells after PMA and ionomycin stimulation compared to media alone (Figure 7B). As expected per previous results the production of IFNγ is reduced by the presence of the CB-PRP derived preparations (~60%), but the reduction is not uniform across preparations (incubation with CB-PR leads to ~50% less INFγ for example) but it is not significantly different within preparations for the different genotypes. In agreement with our previous study, we did detect a difference in reduction of IFN-γ according to genotype with CB-PPP. Samples typed as V homozygous showed less of a reduction in production of IFN-γ (mean 52% ± 2.4 SEM) compared to M homozygous and V/M heterozygous samples (mean 42% ± 1.5 SEM) and was statistically significant (*p* = *0.0074*). Overall, 50% of samples tested were V/V and 50% M/M or M/V genotype.

## Discussion

In this study we have assessed and compared the properties of 3 different CB-PRP preparations, CB-PPP, CB-PL and CB-PR. We measured the concentrations of various factors which are thought to contribute to the activity of CB-PRP. We found that as expected the CB-PPP preparation had lower concentrations of most analytes, while there was less differences between CB-PL and CB-PR. In general, the concentrations of the key analytes were higher in CB-PRP preparations than in tears and comparable to those in adult serum. This is similar to other reported studies (23, 24), though we measured additional, previously not reported analytes.

Further we looked at the immunomodulatory properties of the 3 preparations, by investigating their effects on the activation of NK, NKT, and T cells. We found that all 3 preparations reduced the expression of activation markers NKG2D and CD107a in all cell types investigated to different degrees and further they also decreased the expression of INFγ in NK cells. As we used the same unit to make all three preparations, and the paired and unpaired analysis give almost identical results, it is most likely the effects are due to the PRP preparation and only some of the effect is due to individual variations between individuals. As we have reported previously, we believe this reduction is caused by the presence of soluble NKG2D ligands (MICA, MICB and ULBP1) in the CB-PRP preparations, but it would be prudent to have a larger sample size to confirm this. Studies in tumor biology do corroborate this idea, as soluble NKG2D ligands produced by tumor cells have been reported to downregulate NK cell responses (25, 26).

To understand better the properties of these PRP preparations and the processing methodology, it would be important to measure the concentrations of the GFs at different stages during the processing and see how the different steps affect the final product.

The mechanism of action of CB-PRP in terms of immunomodulation has been investigated by us and others (12, 22). The anti-inflammatory molecule transforming growth factor beta (TGFβ), is an obvious candidate to exert such a function as it is known to be present in fairly high levels in PRP (27–29). Some studies have looked at the effect of TGFβ on the induction of T-regulatory (Tregs) cells as a plausible mechanism to explain the overall anti-inflammatory properties of PRP preparations (7). In contrast, in our previous studies we were able to demonstrate that CB-PRP preparations contain soluble molecules that act as ligands for NK cell receptors and result in reduction of NK cell activation potential (22). These soluble molecules, which have an activating role when expressed on the cell surface, interact with NKG2D and directly down-modulate cytotoxicity of cells bearing this receptor in a dose-dependent manner, independent of a similar effect exerted by TGFβ (12). Further work delineating the differential roles of the various types of NKG2D ligands (NKG2DLs) revealed that soluble ULBP1 (sULBP1) was the most abundant ligand in CB-PRP. Furthermore, we also found that a second NKG2DL termed MICA, also supressed NK function, but this ability was affected by a genetic polymorphism in the *MICA* gene itself (12). A dimorphism in MICA gives rise to two possible variants of the protein with either a methionine (Met) or a valine (Val) at residue 129. This difference has been found to have functional consequences, with the affinity for the receptor being higher on the Met containing protein (14). Indeed, investigations found that PRP preparations from donors who had at least one Met allele were able to down regulate the expression of NKG2D, CD107a, and INFγ in NK cells significantly more than PRP preparations form Val homozygote donors, revealing a functional difference affecting the anti-inflammatory potential of sMICA variants (12).

The use of PRP as a treatment for eye (9) and skin lesions (30) has been established for some time. Though initial studies were carried out mainly on autologous PRP preparations, as a means of avoiding transmission of pathogens, the use of allogeneic PRP has been favored in recent years (9, 31). The use of allogeneic PRP has several advantages, and it offers a means of standardizing the preparation and quality control to make dosing regimens more accurate. Amongst the sources of PRP, CB has been studied closely in the last decade or so, as it has all of the advantages of other allogeneic sources, with the extra advantages of being essentially a waste by product which requires very little extra effort to prepare; having special properties and being less likely to have unexpected transmissible viral pathogens, as mothers are checked during pregnancy.

Currently we are using CB-PRP preparations on two separate clinical trials for two indications: an eye drop treatment for Neurotrophic Keratopathy (NCT03084861) and a gel patch for diabetic foot ulcers (NCT02389010). However, there is a need to establish the likely differences in preparations and how the biochemical content and functional attributes of each preparation may affect their use. We propose that for skin ulcers, the most appropriate preparation would be platelet releasate (PR) in gel form (CB-PG), where the GFs content is released gradually (32). This contains the highest concentrations of both angiogenic as well as trophic and wound healing factors and the lowest concentration of inflammatory molecules. For skin regeneration several phases are required, first there is a vasoconstriction and platelet aggregation phase to stop bleeding, followed by an inflammatory phase which recruits several cells (mainly neutrophils) into the site followed by a proliferative phase where there is new tissue formation (33). The second phase requires pro-inflammatory molecules such as TNFα and INFγ, as well as factors that promote collagen degradation, followed by a re-epithelialisation phase where fibroblasts are recruited to the site and with the help of growth factors induce the multiplication of this fibroblast, which in turn deposit new collagen. In chronic wounds there is a clear disruption to this process therefore providing these factors exogenously should have a positive effect. Consequently, preparations with high levels of TNFα, for example, which increase inflammation, as well as metalloproteinases and their inhibitors, such as the MMPs and TIMPs, which degrade collagen, should aid in the induction of the wound healing cascade (34). High levels of bFGF and EGF consolidate the effect by promoting fibroblast proliferation. The necessary neo vascularisation is also promoted by VEGF, though the levels of this factor are modest in the CB-PR preparation. Importantly the CB-PR preparation reduces the expression of NGK2D activating receptor as well as CD107a in NK cells only modestly, thus the necessary immune cell activation and IFNγ production is not blocked by this preparation. Previous work using platelet lysate preparations in *in vitro* models of wound healing demonstrated that the combination of factors present in platelet lysates was able to promote wound healing in a dose dependent manner (35). CB-PC derivatives (lysate and releasate) are rich in GFs, and are attractive sources for many clinical applications in degenerative conditions, such as joint and cartilage pathologies (36, 37), where injection of this product can be beneficial.

For the treatment of ocular conditions, preparations need to be appropriate to the indication being treated. On the one hand, ocular ulcers and other conditions that have damaged the ocular surface where regeneration is required need special preparations (38). These should contain sufficient quantities of EGF, bFGF, and a certain quantity of inflammatory cytokines to activate the regenerative process. However, they must not promote the excessive proliferation of fibroblasts leading to scarring, as it would impair the function of the cornea. The CB-PL preparation has moderately high levels of EGF, bFGF, HGF, PDGF, and VEGF and fairly high levels of TGFβ (compared to CB-PPP and CB-PR), mostly in line with those found in natural tears, except for VEGF which is much higher in PL. The CB-PL preparation has a dramatic effect on NK, NKT and CD3 T-cells. It reduces the expression of both NKG2D and CD107a substantially indicating a reduction on the activation potential of these cells, thus likely reducing overall inflammation and disease progression but without compromising viability.

For ocular diseases where the corneal surface is not yet compromised, such as dry-eye disease, which is usually caused by a deficiency of the lacrimal functional unit and is accompanied by ocular surface inflammation and irritation due to lack of lubrication (39), the CB-PPP preparation may offer an optimal topic treatment, though here we analyzed the raw material, the topical eye drops used for clinical application are diluted in Plasmalyte, which may dilute the fibrinogen and the pro-thrombin present in the CB-PRP avoiding their possible negative effects. So far, studies have not shown any safety issues caused by the presence of fibrinogen which is present in autologous PRP preparations too (40, 41), however, further studies are necessary to measure this more accurately. This preparation contains low levels of pro-angiogenic and wound healing factors, it also reduces the activation of NKT cells by about 50%, which may arrest the progression of disease caused by the recruitment of Th17 cells. Previous studies in animal models of severe dry eye disease, showed that activated NK and NKT cells up-regulated IL-6 and IL-23 and created an environment where dendritic cells could skew T cell activation to a more pathogenic Th17 phenotype (42). In the absence of NK and NKT cells, disease progression was less severe (43), as no pathogenic Th17 cells were activated (42). Additionally, in the presence of CB-PRP derivatives preparations there is a substantial reduction (~50%) in the production of INFγ by immune cells, which would otherwise contribute to the inflammatory process. The CB-PPP preparation contains the lowest levels of the pro-inflammatory cytokines IL-6, IL-1α, and TNFα, and substantial amounts of soluble NKG2D ligands (ULBP1, MICA, and MICB), making it ideal to treat conditions where inflammation has a major role in disease progression.

In conclusion, CB has unique properties and can be used as a source of plasma and platelet to formulate preparations for regenerative medicine, that can be used for the topical treatment of several eye conditions that affect the ocular surface, such as severe dry eye disease, corneal ulcers and burns. Furthermore, these preparations could also serve as an optimal adjuvant to help in skin wound healing and for articular regenerative processes. Through differential processing of CB-PRP production, we have formulated three blood derived preparations which have different and interesting biochemical and immunomodulatory properties which can be investigated in translational applications. Further work in evaluating the safety and efficacy of these preparations must now be undertaken.

Additionally, future studies should include: (a) *in vivo* safety and efficacy studies in models of corneal and skin wounds. (b) the assessment of vitamin A concentration on final preparations, as it is known to be light sensitive (44), but an essential ingredient in eye drop preparations and (c) the effects of the preparations on the immune response in general.

## Data Availability Statement

All datasets generated for this study are included in the article/Supplementary Material.

## Ethics Statement

Plasma samples from individual CB units were obtained from BioBank (BST). The informed consent for the use of samples for research was obtained in accordance with the requirements of the Declaration of Helsinki and local laws at the time of collection. This study was approved by the BST local Research Ethics Committees (HCB/2017/0785).

## Author Contributions

DS and SC designed, carried out experiments, and wrote the manuscript. DH analyzed results, designed, and wrote manuscript. RC-M design study, analyzed results, and wrote manuscript. SQ, LR, and AM designed study and reviewed the manuscript.

## Conflict of Interest

The authors declare that the research was conducted in the absence of any commercial or financial relationships that could be construed as a potential conflict of interest. The reviewer JB declared a past co-authorship with one of the authors SQ to the handling editor.
