# Supplementary material for: Cord Blood Platelet Rich Plasma Derivatives for Clinical Applications in Non-transfusion Medicine
Source: Front Immunol. 2020 May 27;11:942. doi: 10.3389/fimmu.2020.00942 (PMC7266986; doi:10.3389/fimmu.2020.00942)
Supplement: Supplementary file 1 [file Data_Sheet_1.PDF]

Supplementary Material

1 Supplementary Data

1.1 Supplementary Figures

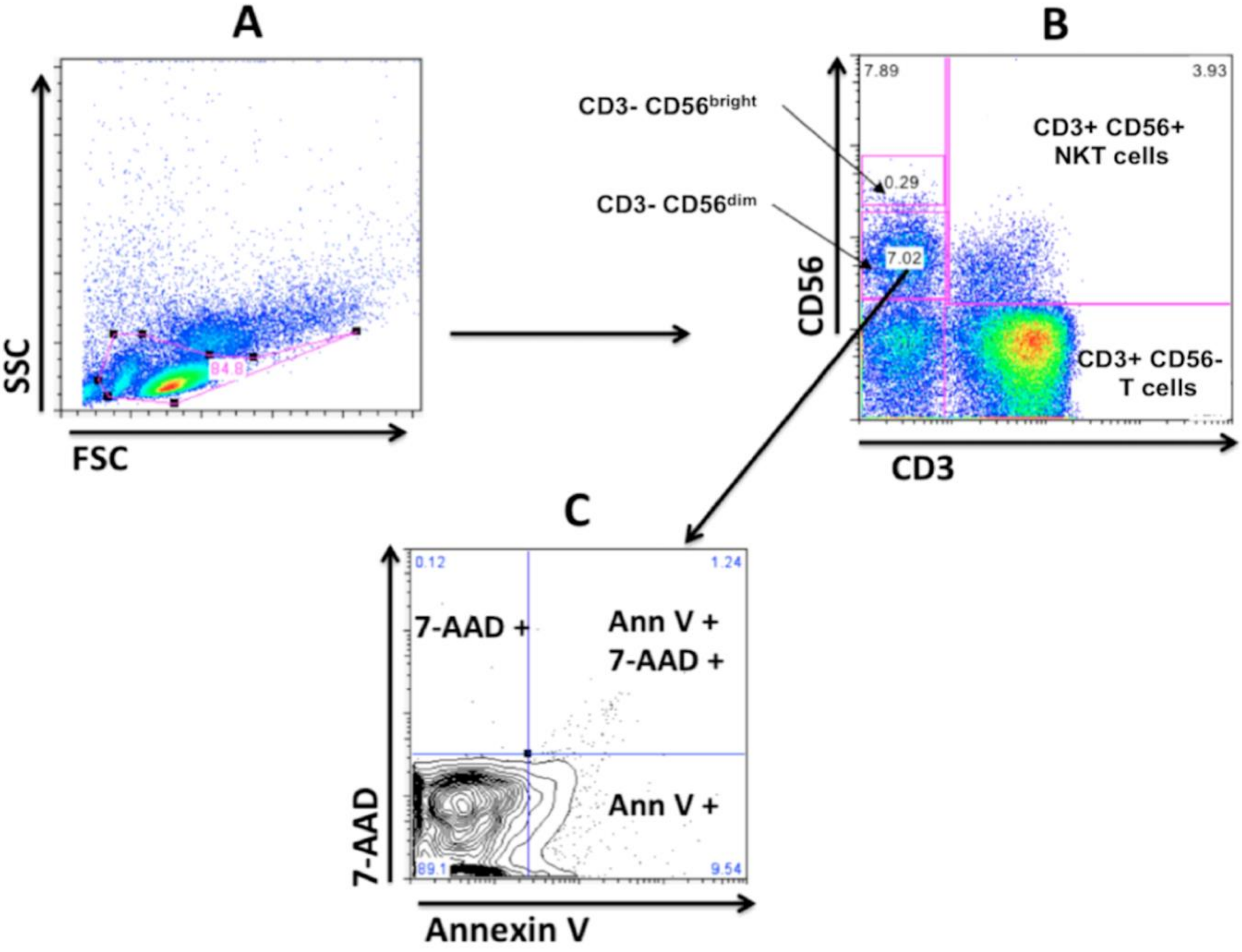

Supplementary Figure S1. Gating strategy to enumerate phenotype and viability of different cell types in PBMCs samples incubated in plasma preparations.

A. Forward and side scatter used for gating of lymphocytes populations

B. NK, NKT and T cells were segregated according to the expression of CD3 (PE) and CD56 (APC) as shown. [NK cells- CD3-CD56$^{dim/bright}$, NKT cells- CD3+CD56+, T cells-CD3+CD56-]

C. Each subpopulation in B was then analysed for cell viability using the 7-AAD fluorescent dye and annexin V (FITC). Viable cells are negative for both 7AAD and Annexin V.

Gates were based on FMO controls (not shown).
